# Health-related Quality of Life among Children Living with Rare Diseases in China: a Nationwide Study

**DOI:** 10.1007/s11482-025-10510-5

**Published:** 2025-11-06

**Authors:** Jiazhou Yu, Huanyu Zhang, Shanquan Chen, Richard H. Xu, Shuyang Zhang, Dong Dong

**Affiliations:** 1 Jockey Club School of Public Health and Primary Care, The Chinese University of Hong Kong, Hong Kong SAR, China; 2 Clinical Big Data Research Center, The Seventh Affiliated Hospital, Sun Yat-sen University, Shenzhen, 518107 China; 3 School of Public Health, The University of Hong Kong, Hong Kong SAR, China; 4 Department of Rehabilitation Sciences, The Hong Kong Polytechnic University, Hong Kong SAR, China; 5 Department of Cardiology, Peking Union Medical College Hospital, Chinese Academy of Medical Science and Peking Union Medical College, Beijing, China; 6 Shenzhen Research Institute, The Chinese University of Hong Kong, Guangdong Shenzhen, China

**Keywords:** Pediatrics, Infants, Wellbeing, Predictors, Asia

## Abstract

A large proportion of rare diseases (RDs) manifest in childhood, posing substantial physical and psychosocial burden on pediatric populations. This study aims to evaluate the health-related quality of life (HRQoL) and associated factors among children living with RDs across key age groups in China. A nationwide cross-sectional survey was conducted on 9,110 infants and children diagnosed with 28 different RDs in China in 2019–2020. The survey collected socio-demographic and disease-related factors of children and family-related factors such as parental wellbeing, and family functioning. HRQoL of children was measured by parent proxy-report version of the Pediatric Quality of Life Inventory™ 4.0 generic core scales or Infant Scales. Multivariable regression analysis was used to identify factors associated with children’s physical, psychosocial, and overall HRQoL. Substantially impaired HRQoL was observed across all domains, particularly for those with Huntington’s disease, mucopolysaccharidosis and Dravet syndrome. Lower HRQoL was associated with older age, male sex, lower socio-economic status, worse parental wellbeing and family functioning, lack of treatment, diagnostic delays, comorbidities, and higher disease severity. The findings suggest that RDs impose substantial negative impacts on Chinese children’s physical and psychosocial wellbeing. Efforts are needed to improve diagnosis and treatment accessibility. Targeted psychosocial support and family-centered strategies are needed to mitigate burdens on this vulnerable group.

**Supplementary Information:**

The online version contains supplementary material available at 10.1007/s11482-025-10510-5.

## Introduction

Rare diseases (RDs) are conditions that affect a small number of individuals compared to more common illnesses. It is defined as fewer than 200,000 people in the United States (Commissioner, 2022) and fewer than 5 in 10,000 in the European Union (European Commission, n.d.). Although individually rare, RDs collectively affect millions of people worldwide. Approximately 70% of RDs manifest in childhood and often involve complex physical, cognitive, and developmental challenges (The Lancet Diabetes Endocrinology, 2019; Nguengang Wakap et al., 2020). Many children with RDs live with ongoing disability, delayed diagnosis, and limited treatment options (Bavisetty et al., 2013; Marimpietri & Zuccari, 2023; Witt et al., 2023), creating a significant burden on their physical health, emotional wellbeing, daily functioning, and family life (Schieppati et al., 2008; Verhoof et al., 2012).

Understating the full impact of these conditions requires a holistic perspective. Health-related quality of life (HRQoL) provides such a framework by capturing an individual’s subjective perception of their wellbeing across physical, emotional, and social domains (Hays & Reeve, 2008). In contrast to clinical assessments that focus primarily on symptoms, HRQoL integrates the psychosocial aspects of a patient’s experience. Grounded in theoretical frameworks like the Wilson and Cleary model, which links biology, symptoms, functioning, and environmental factors to overall health outcomes (Wilson & Cleary, 1995), HRQoL research provides critical insights into how children adapt to the challenges of living with an RD.

Research from different settings consistently shows that children with RDs report lower HRQoL than their peers in the general population (Gao et al., 2020a; Ries et al., 2005; Thomas et al., 2006; Yamaguchi et al., 2017), especially in the domain of physical functioning (Arrington-Sanders et al., 2006; Britto et al., 2004; Palermo et al., 2002). The majority of literature come from Western settings, where the psychosocial impact on the child is a key theme (Adama et al., 2023). For example, a study from Australia reported that around half of children with rare diseases experienced mental difficulties at school (Belzer et al., 2022). There is also an emphasis on the economic and emotional burden on caregivers which in turn affects child HRQoL(Buckle et al., 2024; Domaradzki et al., 2025; Dumbuya et al., 2025). Evidence from Asia, while growing, primarily focuses on the impact of socioeconomic factors and treatment availability on HRQoL (Gao et al., 2020b; Ng et al., 2022; Qi et al., 2021a; Zhu et al., 2025a). For instance, a nationwide study from China identified poverty and diagnostic delay as key contributors to disease burden encompassing impaired HRQoL (Yu et al., 2024). These focuses likely reflect the relatively limited medical resources and support systems in Asian regions, which may heighten the burden of RDs (Rodrigues et al., 2025). Emerging evidence indicates that cultural norms around family roles, child autonomy, and emotional expression influence children’s HRQoL (Kang et al., 2024; Mousavi et al., 2019; Satriono et al., 2024; Zhu et al., 2025b). Together, these findings suggest the importance of evaluating HRQoL within specific healthcare and cultural context to inform targeted support and policy.

The impact of rare diseases on children extends to families, as parents and caregivers often report increased stress and financial strain that compromised their own quality of life (Domaradzki et al., 2025; Valcárcel-Nazco et al., 2022; Witt et al., 2023). Theoretical models such as family systems theory demonstrated that illness is not an individual event but a systemic stressor that reorganizes roles, rules, and relationships across the entire family (Dore, 2008). While family functioning is known to influence child HRQoL in other chronic conditions (Herzer et al., 2010; Szyndler et al., 2005; Yang et al., 2021), its specific role within the unique context of pediatric rare diseases remains underexplored. Moreover, there is a significant research gap exists concerning the HRQoL of infants with RDs, despite infancy being a critical period of development. Addressing these gaps is essential for developing targeted interventions that support the entire family unit from the earliest stages.

In China, an estimated 16.8 million individuals are living with RDs, including 11.8 million children, posing significant burdens on healthcare system and society (Li et al., 2021). However, despite the efforts in prioritizing new drugs for RDs and children in review and approval, China has not yet passed any legislations specifically addressing RDs or orphan drugs like the Orphan Drug Act enacted in the US which outlines the definition of RDs, establishes drug designation system, and provides R&D incentives (Ying et al., 2021). Studies have revealed high misdiagnosis rates (Qi et al., 2021b) and undermined psychological wellbeing (Chen et al., 2022) among Chinese children and adolescents living with RDs. Moreover, consistent with global trends, research in China that includes very young children or investigates family wellbeing alongside child HRQoL remains scarce.

This study aims to evaluate the HRQoL of children with various RDs in China, from infancy through adolescence. The study also examines the association of clinical factors, family functioning, and parental wellbeing with child HRQoL. By adopting a family-centered, quality-of-life perspective, this research seeks to generate context relevant evidence to inform targeted interventions and enhance the understanding of the lived experiences of this vulnerable population.

## Methods

### Study design and data collection

This study is a nationwide cross-sectional survey. Given the lack of sampling frame of individuals with RDs in China, the study employed a non-probability sampling approach. The participants were recruited from registered individuals with 32 different national RD patient organizations, representing 33 different RDs included in China’s First National List of Rare Disease published in 2018. Data were collected between August 2019 and January 2020. The process of questionnaire design and data collection, and quality control has been described in detail elsewhere (Xu et al., 2023).

In brief, two versions of questionnaire, self-report and proxy-report versions, were distributed on an online platform. Participants under the age of 18 were required to complete the proxy-version by a parent or legal guardian. All participants were required to read the consent form and click on “consent to participate” before able to enter the study. The survey collected a total of 20,804 responses.

In this analysis, responses were excluded if: (1) the participant was aged 18 or above at the time of survey (*n* = 7,424); (2) responses on participant age or HRQoL variables were not complete (*n* = 2,754); (3) the report was proxy competed by a caregiver other than parents (*n* = 1,515). Finally, a total of 9,110 participants under the age of 18 were included in the analysis. In this analysis, we used the data on: (1) socio-economic background of the child (e.g., age, sex, household income); (2) diagnosis and treatment of the child (e.g., onset of symptoms, treatment status, disease category); (3) background of parents and family functioning (e.g., education, marital status, wellbeing).

The ethical approval of this study was obtained from the Survey and Behavioural Research Ethics Committee of [blinded for review].

### Pediatric health-related quality of life

Pediatric Quality of Life Inventory™ (PedsQL™) is one of the most widely used HRQoL instruments for measuring HRQoL for children and adolescents (Varni et al., 2003a). In this study, the parent-proxy version of PedsQL™ 4.0 generic core scales and PedsQL™ Infant Scales was completed.

HRQoL of children aged 2–17 was measured by PedsQL™ 4.0 generic core scales, which was designed for healthy children as well as children with a wide range of health conditions (Varni et al., 1999). Parent-report of PedsQL™ 4.0 generic core scales includes four forms, respectively designed for ages 2–4 (toddlers), 5–7 (young children), 8–12 (children), and 13–18 years (teens). The scales consists of two subscales: Physical Health and Psychological Health (covering Emotional, Social, and School Functioning), and has shown good validity and reliability (Varni et al., 2006). The items of each version are essentially identical but designed or worded differently for developmentally appropriate language except that version for toddlers included only 3 items for School Functioning Scale (Varni et al., 2003a). The Chinese Version of PedsQL 4.0 generic core scales has been validated (Ji et al., 2011).

HRQoL of infants aged 1–24 months was measured by PedsQL™ Infant Scales (Varni et al., 2011). Infant scales include two versions designed respectively for infant aged 1–12 and 13–24 months, measuring Physical Health (covering Physical Functioning, Physical Symptoms), and Psychosocial Health (covering Emotional, Social, and Cognitive Functioning). The Cronbach’s alpha ranged for overall scales and subscales ranged from 0.91 to 0.97 (Supplementary Table 1), indicating excellent internal consistency reliability in our study sample (George & Mallery, 2005).

**Table 1:** Characteristics of included children (*n* = 9,110)

Variables	*n* (%)	Physical HRQoL		Psychosocial HRQoL		Overall HRQoL	
		Mean (SD)	*p*-value	Mean (SD)	*p*-value	Mean (SD)	*p*-value
Overall		58.61 (26.70)		58.23 (22.13)		58.35 (22.36)	
Sex			< 0.001		< 0.001		< 0.001
Male	5776 (63.4%)	55.57 (26.70)		56.77 (21.81)		56.29 (22.12)	
Female	3334 (36.6%)	63.88 (25.89)		60.76 (22.46)		61.94 (22.33)	
Age of child, years			< 0.001		< 0.001		< 0.001
<2	1817 (20.0%)	64.08 (18.21)		63.06 (18.86)		63.49 (17.47)	
2–4	2561 (28.1%)	62.43 (28.55)		60.89 (23.93)		61.50 (24.71)	
5–17	4732 (51.9%)	54.44 (27.71)		54.94 (21.75)		54.68 (22.04)	
Household registration			< 0.001		< 0.001		< 0.001
Rural	5018 (55.1%)	57.39 (26.52)		57.22 (22.2)		57.27 (22.29)	
Urban	4092 (44.9%)	60.1 (26.86)		59.47 (21.99)		59.68 (22.37)	
Parental education			< 0.001		< 0.001		< 0.001
At least one parent below secondary	4179 (46.0%)	55.7 (26.7)		56.12 (22.18)		55.95 (22.25)	
Both secondary or above	4899 (54.0%)	61.13 (26.44)		60.04 (21.92)		60.43 (22.23)	
Annual household income			< 0.001		< 0.001		< 0.001
< CNY¥50,000	3847 (42.2%)	56.79 (26.58)		56.46 (22.28)		56.6 (22.32)	
CNY¥50,000–100,000	3648 (40.0%)	58.84 (26.63)		58.49 (21.9)		58.59 (22.23)	
>CNY¥100,000	1615 (17.7%)	62.41 (26.76)		61.86 (21.85)		62.01 (22.28)	
Parental marital status			0.14		< 0.001		0.006
Married	8102 (89.2%)	58.77 (26.64)		58.52 (22.07)		58.6 (22.29)	
Divorced/Separated/Widowed	985 (10.8%)	57.45 (27.24)		56.02 (22.54)		56.53 (22.86)	
Parental HRQoL, mean (SD)	45.60 (20.60)	——		——		——	
Family Functioning, mean (SD)	47.40 (21.69)	——		——		——	
Dependence on assistive tools			< 0.001		< 0.001		< 0.001
None	6485 (71.2%)	67.12 (22.89)		64.01 (20.44)		65.21 (19.71)	
Mild	1669 (18.3%)	40.62 (22.88)		44.78 (19.03)		43.13 (18.76)	
Substantial	955 (10.5%)	32.28 (24.1)		42.51 (20.32)		38.4 (19.85)	
Active treatment			< 0.001		< 0.001		< 0.001
No	2229 (24.5%)	50.91 (27.66)		54.21 (22.55)		52.87 (22.91)	
Yes	6881 (75.5%)	61.1 (25.9)		59.53 (21.84)		60.13 (21.89)	
Diagnostic delay			< 0.001		< 0.001		< 0.001
<1 year	7978 (88.1%)	60.56 (26.11)		59.86 (21.68)		60.12 (21.82)	
1–2 years	839 (9.3%)	44.71 (26.93)		47.3 (22.3)		46.19 (22.58)	
≥3 years	236 (2.6%)	42.79 (25.14)		42.29 (19.85)		42.35 (19.79)	
Comorbidity			< 0.001		< 0.001		< 0.001
None	5894 (64.7%)	64.04 (25.72)		64.37 (20.32)		64.26 (20.77)	
1	1869 (20.5%)	53.88 (25.06)		52.69 (20.01)		53.08 (20.32)	
≥2	1347 (14.8%)	41.39 (24.5)		39.06 (19.39)		39.86 (19.69)	
Recent diagnosis			0.15		< 0.001		0.001
No	6260 (68.7%)	58.33 (28.56)		57.61 (22.94)		57.86 (23.46)	
Yes	2850 (31.3%)	59.21 (22.09)		59.6 (20.19)		59.44 (19.67)	
Disease category			< 0.001		< 0.001		< 0.001
Endocrine, nutritional and metabolic diseases	4262 (46.8%)	70.31 (23.83)		65.87 (20.87)		67.59 (20.3)	
Diseases of musculoskeletal system	1925 (21.1%)	41.97 (24.01)		50.5 (19.87)		47.19 (19.84)	
Congenital malformations, deformations and chromosomal abnormalities	1930 (21.2%)	48.68 (24.41)		47.14 (20.79)		47.6 (20.61)	
Others	993 (10.9%)	59.95 (22.65)		61.99 (20.01)		61.26 (19.72)	

CNY¥1=USD$0.14

For every child, summary scores were computed for Physical Health subscale (Physical HRQoL), Psychosocial Health subscale (Psychosocial HRQoL), and overall scale (Overall HRQoL), calculated by the sum of all the items over the number of items answered on the subscale/overall scale. The scores range between 0 and 100, with a higher score indicating better HRQoL.

### Socio-demographic and disease-related variables

We collected socio-demographic information on child’s age, rurality of household registration (rural, urban), parental education attainment (at least one parent with education below secondary, both parents with secondary education or above), annual household income (< CNY¥50,000, CNY¥50,000–100,000, >CNY¥100,000) (CNY¥1 = USD$0.14), parental marital status (married, divorced/separated/widowed), parental wellbeing, and family functioning. PedsQL™ 2.0 Family Impact Module is a 36-item parent-reported instrument to measure the impact of pediatric chronic condition on parent HRQoL (covering Physical, Emotional, Social, Cognitive Functioning subscales) and family functioning (covering Daily Activities and Family Relationship subscales) (Chen et al., 2011; Varni et al., 2004). In our study, the summary scores of parent HRQoL and family functioning were used, with total score ranging between 0 and 100 and higher scores indicating better parental wellbeing and family functioning, respectively. Rare disease-related variables included the child’s dependence on assistive devices (none, mild, substantial), treatment status (active, non-active), diagnostic delay (< 1 year, 1–2 years, ≥ 3 years), comorbidity (none, 1, ≥ 2), recently diagnosed defined by being diagnosed in the past year (no, yes), and disease category (Endocrine, nutritional and metabolic diseases, Disease of musculoskeletal system, Congenital malformations, deformations, Others).

### Statistical analysis

Socio-demographic and disease-related variables were described in number and percentages for categorical variables and in mean and standard deviation (SD) for continuous variables. Physical HRQoL and Psychosocial HRQoL were described by background characteristics in mean and SD and compared with ANOVA.

Univariable and multivariable linear regression analyses were conducted to identify factors associated with physical, psychosocial, and overall HRQoL of children. The explanatory factors included sex and age of the child, parental education, annual household income level, parental wellbeing, family functioning, dependence on assistive devices, treatment status, diagnostic delay, comorbidity, and disease category. We conducted three sets of regression models with Physical HRQoL, Psychosocial HRQoL, and Overall HRQoL as the outcome, respectively. Variables that were significant at *p* < 0.10 level in univariable regression were further included in the multivariable models. We evaluated multicollinearity using variance inflation factors (VIFs), with VIF greater than 5 indicating detection of multicollinearity (Kutner, 2005). To address the potential shared variance between parental wellbeing and family functioning, we performed sensitivity analyses in which these variables were included individually in the models. Considering that developmental characteristics and disease profiles may differ between infants and older children, conducted subgroup analyses to examine potential differences in factors associated with HRQoL. The statistical significance is set at *p* < 0.05. All the analyses were conducted on Stata 14.0.

## Results

### Descriptive characteristics

A total of 9,110 children with 28 different RDs were included in this study, including 1,817 infants and 7,293 school-aged children and adolescents. The descriptive characteristics of included children are described in Table 1. Approximately two-thirds of the children were boys (63.4%). More than half (51.9%) of the children aged between 5 and 17 years, 28.1% aged between 2 and 4 years, and 20.0% were below the age of two. Regarding parental characteristics, 54.0% of the children had both parents with secondary education or above, and 10.8% reported divorcer/separated/widowed parents. The mean score of parent HRQoL and family functioning was 45.60 and 47.40, respectively. The majority of children were independent of assistive devices (71.2%), under active treatment (75.5%), diagnosed within one year after symptom onset (88.1%). Respectively 46.8%, 21.1%, 21.2%, 10.9% of children had endocrine, nutritional, and metabolic diseases, diseases of musculoskeletal systems, congenital malformations, deformations, and chromosomal abnormalities, and other RDs. The distribution of 28 diseases is described in Supplementary Table 2.

**Table 2:** Multivariable linear regression to identify factors associated with physical, psychosocial, and overall PedsQL scores (*n* = 9,110)

Variables	Physical HRQoL		Psychosocial HRQoL		Overall HRQoL	
Sex	*Coeff (95% CI)*	*p-value*	*Coeff (95% CI)*	*p-value*	*Coeff (95% CI)*	*p-value*
Male	Ref.		Ref.		Ref.	
Female	3.24 (2.37, 4.11)	< 0.001	1.54 (0.83, 2.25)	< 0.001	2.20 (1.52, 2.89)	< 0.001
Age of child, years						
<2	Ref.		Ref.		Ref.	
2–4	−2.56 (−3.75, −1.37)	< 0.001	−3.44 (−4.41, −2.48)	< 0.001	−3.10 (−4.03, −2.17)	< 0.001
5–17	−6.92 (−8.03, −5.81)	< 0.001	−7.77 (−8.66, −6.88)	< 0.001	−7.55 (−8.42, −6.68)	< 0.001
Household registration						
Rural	——		Ref.		Ref.	
Urban	——		0.91 (0.25, 1.57)	0.007	0.83 (0.11, 1.55)	0.025
Parental education						
At least one parent below secondary	Ref.		——		Ref.	
Both secondary or above	2.14 (1.24, 3.05)	< 0.001	——		0.99 (0.26, 1.73)	0.008
Annual household income						
>CNY¥100,000	Ref.		——		——	
CNY¥50,000–100,000	1.07 (0.13, 2.01)	0.026	——		——	
< CNY¥50,000	1.71 (0.43, 2.99)	0.009	——			
Parental HRQoL	0.24 (0.21, 0.27)	< 0.001	0.30 (0.27, 0.32)	< 0.001	0.28 (0.25, 0.30)	< 0.001
Family Functioning	0.10 (0.07, 0.13)	< 0.001	0.14 (0.12, 0.17)	< 0.001	0.13 (0.11, 0.15)	< 0.001
Dependence on assistive devices						
None	Ref.		Ref.		Ref.	
Mild	−16.03 (−17.15, −14.91)	< 0.001	−10.16 (−11.07, −9.25)	< 0.001	−12.45 (−13.33, −11.57)	< 0.001
Substantial	−22.55 (−23.97, −21.14)	< 0.001	−11.36 (−12.51, −10.21)	< 0.001	−15.80 (−16.91, −14.69)	< 0.001
Treatment						
Active	Ref.		Ref.		Ref.	
Non-active	−3.69 (−4.66, −2.71)	< 0.001	−1.84 (−2.62, −1.05)	< 0.001	−2.62 (−3.38, −1.86)	< 0.001
Diagnostic delay						
None	Ref.		Ref.		Ref.	
1–2 years	−5.12 (−6.55, −3.70)	< 0.001	−4.33 (−5.49, −3.18)	< 0.001	−4.76 (−5.88, −3.65)	< 0.001
≥3 years	−5.06 (−7.64, −2.48)	< 0.001	−6.16 (−8.25, −4.07)	< 0.001	−5.65 (−7.67, −3.63)	< 0.001
Comorbidity						
None	Ref.		Ref.		Ref.	
1	−3.86 (−4.92, −2.81)	< 0.001	−5.88 (−6.73, −5.03)	< 0.001	−5.15 (−5.97, −4.33)	< 0.001
≥2	−9.54 (−10.79, −8.29)	< 0.001	−13.19 (−14.20, −12.17)	< 0.001	−11.85 (−12.83, −10.86)	< 0.001
Disease category						
Endocrine, nutritional and metabolic diseases	Ref.		Ref.		Ref.	
Diseases of musculoskeletal system	−15.32 (−16.51, −14.14)	< 0.001	−5.24 (−6.19, −4.29)	< 0.001	−9.01 (−9.93, −8.09)	< 0.001
Congenital malformations, deformations and chromosomal abnormalities	−11.26 (−12.4, −10.12)	< 0.001	−8.63 (−9.55, −7.71)	< 0.001	−9.68 (−10.56, −8.79)	< 0.001
Others	−5.71 (−7.11, −4.3)	< 0.001	−0.24 (−1.37, 0.9)	0.008	−2.22(−3.32, −1.13)	< 0.001

CNY¥1 = USD$0.14. —— Non-significant association

**Fig. 1:**
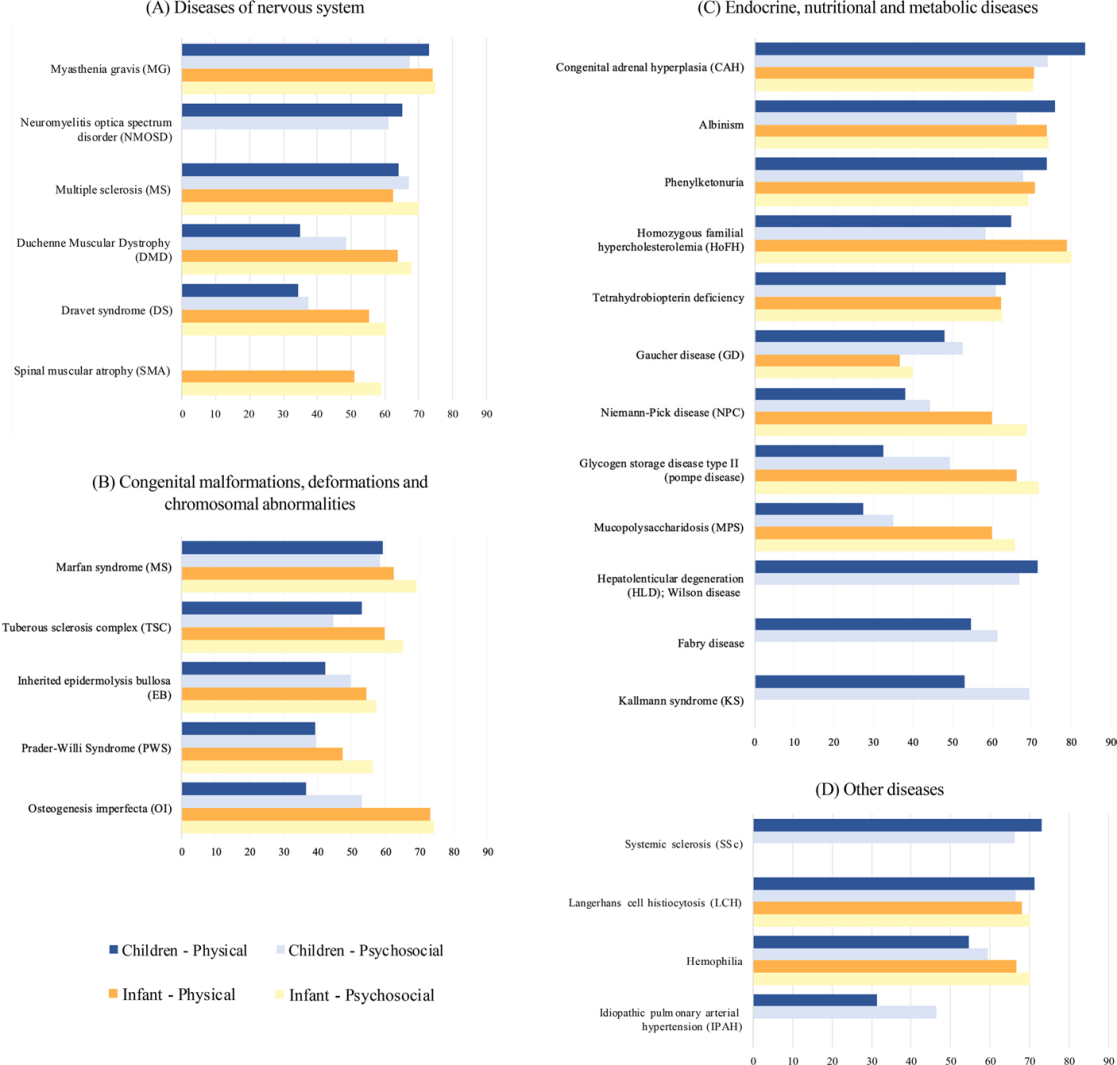
Physical and psychosocial HRQoL scores among children aged 2–17 years and infants aged 1–23 months, by disease category: (**A**) Diseases of nervous system; (**B**) Congenital malformations, deformations and chromosomal abnormalities; (**C**) Endocrine, nutritional and metabolic diseases; (**D**) Other diseases

The mean for Physical HRQoL, Psychosocial HRQoL, and Overall HRQoL was 64.08 (SD 18.21), 63.06 (SD 18.86), and 63.58 (SD17.47) for infants, and 57.25 (SD 28.27), 57.03 (SD 22.72), and 57.07 (SD 23.24) for children and adolescents aged 2–17 years. The Overall HRQoL and Psychosocial HRQoL significantly differed by all background characteristics (p-values ≤ 0.001) while the Physical HRQoL significantly differed by all variables except for parental marital status (*p* = 0.15). By disease, the mean Overall HRQoL ranged between 19.44 (SD 20.97) for Huntington’s Disease to 74.53 (SD 16.08) for congenital adrenal hyperplasia (Supplementary Table 2). The Physical and Psychosocial HRQoL are described separately for infants and older children by diseases in Fig. 1.

### Factors associated with HRQoL

The results of multivariable regression models are described in Table 2. All VIFs were lower than 2.5, suggesting no problematic multicollinearity (VIF > 5) (Kutner, 2005). Older age [β=−6.92 for age 5–17 years, β=−2.75 for age 2–4 years], higher dependence on assistive devices [β=−22.55 for substantial dependence; β=−16.03 for mild dependence], non-active treatment [β=−3.69], longer diagnostic delay [β=−5.06 for ≥ 3 years], comorbidities [β=−9.54 for ≥ 2 comorbidities], and having diseases other than endocrine, nutritional and metabolic diseases, particularly diseases of musculoskeletal system [β=−15.32] were significantly associated with lower Physical HRQoL of children. Female sex [β = 3.23], parental education [β = 2.14], household income [β = 1.71 for high level], parental wellbeing [β = 0.24], and family functioning [β = 0.10] were positively associated with the score.

Older age [β=−7.77 for age 5–17 years; β=−3.44 for age 2–4 years], higher dependence on assistive devices [β=−11.36 for substantial dependence; β=−10.16 for mild dependence], longer diagnostic delay [β=−6.16 for ≥ 3 years], more comorbidities [β=−13.19 for ≥ 2 comorbidities], and having disease of musculoskeletal system [β=−5.24] were significantly associated with lower Psychosocial HRQoL. Female sex, [β = 1.54], urban household registration [β = 0.92], better parental wellbeing [β = 0.30] and family functioning [β = 0.14] were associated with higher score.

Similar to factors identified above, the overall HRQoL reflected by Overall HRQoL was associated with sex, age, household registration, parental education, parental wellbeing, family functioning, dependence on assistive devices, treatment status, diagnostic delay, and comorbidities. Those with diseases of musculoskeletal system and congenital malformations, deformations, and chromosomal abnormalities showed significantly lower HRQoL than those with endocrine, nutritional, and metabolic diseases.

### Sensitivity analysis and subgroup analysis

Models including parental HRQoL (Supplementary Table 3) and family functioning (Supplementary Table 4) separately yielded associations consistent with the main analysis, supporting the robustness of our findings despite potential shared variance between the two variables.

The subgroup analysis (Supplementary Table 5) showed that older children and adolescents had lower physical and psychosocial HRQoL while younger infants showed lower psychosocial HRQoL. Parental education level, treatment status, and diagnostic delay only showed significant associations among those aged 2–17 years. Parental wellbeing, family functioning, dependence on assistive devices, comorbidities, and disease category showed similar association on both subgroups with the main analysis.

## Discussion

### Principal findings

Using a HRQoL framework that integrates physical and psychosocial domains, this study has revealed severely impaired wellbeing among children living with 28 different RDs in China. The mean of parent proxy-reported PedsQL score for children and adolescents aged 2–7 years was 57.07, lower than that measured among their counterparts who were healthy (mean 80.74) (Ji et al., 2011) or affected by other common chronic conditions such as cancer (mean 66.54) (Ji et al., 2011), epilepsy (mean 60.18), immune thrombocytopenia (mean 85.10) (Zhang et al., 2016). The score was averaged at 63.49 for infants aged 1–23 months, indicating poorer HRQoL compared to healthy Chinese infants (mean 89.68) (Wang et al., 2017). Generally similar impairment on physical and psychosocial aspects was recorded. Male sex, older age, rural household registration, lower parental education, worse parental wellbeing and family functioning, lack of treatment, diagnostic delays, comorbidities, and higher level of disease severity were significantly associated with undermined pediatric HRQoL. Of 28 different diseases, young children affected by progressive diseases that may demonstrate developmental delay and cognitive impairment (e.g., Gaucher diseases, Dravet Syndrome) showed the lowest HRQoL.

### Factors associated with HRQoL

This study identified factors associated with HRQoL of children with rare diseases. Several factors exceeded the minimal clinically important difference (MCID) thresholds of 4.50 for total score, 6.92 for physical health, and 5.49 for psychosocial health (Varni et al., 2003b), including diagnostic delay, dependence on assistive device, and multiple comorbidities. These findings indicate that disease-related factors may pose the greatest influence on pediatric HRQoL in rare diseases. Targeting and managing these factors could translate to clinically meaningful improvements in affected children. Several socio-demographic factors, such as sex, parental education, and household income, showed statistically significant association but with effect sizes below the MCID thresholds. Although this indicates limited clinical relevance at an individual level, the observed associations still point to social gradients that inform mechanism of pediatric HRQoL that could inform population-level interventions. For example, higher socioeconomic status may facilitate access to medical resources and disease-related knowledge that helps improve children’s HRQoL. The gender-specific differences potentially reflects that girls’ faster developmental trajectories and greater emotional expressiveness may support socio-emotional support and coping with RDs (Chu, 2014). Consistent with prior evidence (Szyndler et al., 2005), family functioning was associated with pediatric HRQoL; we further identified parental wellbeing as a significant contributor, and the magnitude of the child’s HRQoL changes by 28% of the change in parental HRQoL, after controlling for other significant factors. This reflects that the pediatric HRQoL relies considerably on family-related factors. Evidence has suggested that family dynamics, including emotional support, communication, and caregiving, are essential in promoting a child’s psychological and physical well-being, thereby enhancing their HRQoL (An et al., 2024; Feinberg et al., 2022). Conversely, family stressors, such as parental mental health challenges or financial instability, can negatively affect the child’s HRQoL (Buckle et al., 2024; Domaradzki et al., 2025; Dumbuya et al., 2025). In China where family plays a central role, these factors may have a heightened impact, as family members are often the primary sources of support, advocacy, and care for the child (Chen, 2025). After stratified by age group, older age, treatment status, and diagnosis timeline were associated with lower HRQoL only among children and adolescents, which reflects increased burden as disease progresses with age. On the other hand, infants who were older showed better psychosocial HRQoL. This can be explained by the increased support from parents as caregiving burden alleviated with infancy development as well as psychosocial adaption of parents over time.

It has been previously noted that HRQoL is closely linked to the severity of diseases (Krab et al., 2009). In our study, children with musculoskeletal diseases showed particularly impaired physical health, while those with congenital malformations, deformations and chromosomal abnormalities had greater psychosocial health impairment, both far exceeding MICD thresholds. Specifically, among children and adolescents, the lowest HRQoL was observed in Huntington’s disease (HD), mucopolysaccharidosis (MPS), and Dravet syndrome (DS). Among infants, Gaucher disease (GD), Prader-Willi syndrome (PWS), and DS showed the lowest HRQoL. HD, MPS, and DS involve severe cognitive, movement, and psychiatric symptoms (Letort & Gonzalez-Alegre, 2013; Somanadhan & Larkin, 2016), and GD and PWS lead to developmental delays and physical disabilities (Daykin et al., 2021; Cassidy & Driscoll, 2009), which can significantly affect the patient’s physical and mental abilities. The lack of effective and affordable disease-modifying therapies imposes additional physical burden on affected individuals and financial strain on their families, in turn affecting the overall psychosocial wellbeing.

### Contributions to HRQoL research and theory

This study contributes to the field of HRQoL research in several aspects. First, the study extended HRQoL research to pediatric rare disease across full early-life span. By including both infants and children/adolescents within a generic HRQoL model, the study broadened the scope of prior work which has largely excluded infant and showed novel evidence on age-specific patterns. Second, the study demonstrated that in addition to biological and clinical status, contextual and family factors make significant contribution to a child’s overall wellbeing. These finding support the perspective that HRQoL is an outcome of an interplay of clinical, functional, and environmental determinants, as suggested by Wilson and Cleary (Wilson & Cleary, 1995). Third, our findings identified clinically meaningful contributors (diagnostic delay, disease severity, and comorbidities) within a HRQoL framework. In this specific vulnerable population, biological and clinical factors, which are foundational elements in pathway of the Wilson-Cleary model, can substantially shift HRQoL. Moreover, the study integrated parent-child dynamics into pediatric HRQoL, showing that parental wellbeing and family functioning play important roles in shaping children’s experience of and adaption to illness. This aligns with the family-system theory and is particularly relevant in Asian settings where collectivist cultural values around family roles and caregiving are deeply rooted (DeLambo et al., 2011). Lastly, by considering diverse RD collectively, the study supports the universal applicability of a common HRQoL framework across heterogeneous conditions, pointing to shared mechanisms despite etiologic differences.

### Clinical and policy implications

These findings generated valuable implications for practice and policy-making. First, continuous efforts are needed to increase the availability and accessibility of RD diagnosis and treatment in pediatrics. This can be achieved through strategies such as incentivizing drug development for pediatrics, improving diagnostic technique and knowledge, and expanding insurance coverage. Clinicians should proactively identify and manage children with comorbidities. Additional financial support should be considered for families from lower socio-economic backgrounds. Priority in these efforts should be given to diseases with particularly high health burden such as HD, GD, and DS. Second, a family-centered approach should be adopted, incorporating genetic counselling, psychological interventions, and social support for both affected children and their parents. Third, educational support is essential for children and adolescents. Specialized educational and social integration strategies should be implemented to accommodate their needs in schools and communities. Lastly, it is critical to equip parents with disease-related knowledge and psychological support from the early stages. Establishing peer groups can be an effective approach for accessing information and social support. These psychosocial and educational support measures can be considered as primary management strategy for diseases with relatively lower health burden such as congenital adrenal hyperplasia, phenylketonuria, and albinism.

### Limitations

Due to the lack of a complete sampling frame of individuals with RD in China, this study employed a non-probability sampling approach which may introduce selection bias. The causal relationship between the examined factors and HRQoL cannot be established given the cross-sectional study design. While HRQoL outcomes may differ by context and local policies, we believe that our findings and implications have some transferability to other settings, given the shared challenges faced by the pediatric RD populations. While parent proxy-report is commonly used for younger children and considered essential when children are unable to self-report due to young age, cognitive limitations, or illness (Varni et al., 2007), it carries measurement constraints intrinsic to the instrument. As parents tend to rate their children’s HRQoL more negatively than children do themselves (De Clercq et al., 2025), the HRQoL scores reported in this study may be lower than children’s actual self-perceptions. Additionally, the shared informant effect, where both the parent and child are involved in the same family system, could inflate associations between parental and child HRQoL.

### Future research

Building on evidence that biological, functional, and environmental factors contribute to HRQoL, future studies should apply mediation and, where appropriate, moderation analyses to examine directional pathways proposed in the Wilson-Cleary model (Wilson & Cleary, 1995). Further investigation into family functioning and parental well-being is warranted to better understand the psychosocial mechanisms linking family context to child HRQoL. Longitudinal studies are needed to track HRQoL trajectories over time and to evaluate the effectiveness of interventions, including whether multicomponent strategies yield additive benefits. Finally, incorporating both parent-proxy and child self-reports will provide a more comprehensive assessment of children’s HRQoL.

## Conclusion

This study reveals significant burden of rare diseases on the physical and psychosocial HRQoL of infants and young children in China. By including a wide age range and diverse conditions, it addresses a critical evidence gap, particularly in the context of low- and middle-income countries where access to treatment and support services are relatively limited. The finding that both child and family factors, such as disease severity, parental wellbeing, and family functioning, are associated with pediatric HRQoL reinforces the importance of a family-centered approach in pediatric care. These findings point to an urgent need for improved access to diagnosis, treatment, and psychosocial support, especially for children with severe or progressive conditions. Overall, this study provides new evidence on pediatric HRQoL in the context of rare diseases and offers practical insights for research, clinical care, and policy targeting this vulnerable population.

## Supplementary Information

Below is the link to the electronic supplementary material.

Supplementary Material 1 (DOCX. 55.9 KB)

## Data Availability

The datasets used and/or analyzed during the current study are available from the corresponding author on reasonable request. Health-related quality of life among children living with rare diseases in China: a nationwide study.
