# Supplementary material for: Health-related Quality of Life among Children Living with Rare Diseases in China: a Nationwide Study
Source: Appl Res Qual Life. 2025 Nov 6;21(3):1127–45. doi: 10.1007/s11482-025-10510-5 (PMC13452757; doi:10.1007/s11482-025-10510-5)
Supplement: Supplementary file 1 [file 11482_2025_10510_MOESM1_ESM.docx]

**Supplementary Table 1.** Cronbach’s alpha of overall scales and subscales in the study sample.

| Scale | Overall scale | Physical Health subscale | Psychosocial Health subscale |
| --- | --- | --- | --- |
| PedsQL ^TM^ Infant Scale (1-12 Months) | 0.9612 | 0.9248 | 0.9455 |
| PedsQL ^TM^ Infant Scale (13-24 Months) | 0.9696 | 0.9178 | 0.9640 |
| PedsQL ^TM^ Genetic Core Scales for Toddlers (ages 2-4) | 0.9639 | 0.9438 | 0.9485 |
| PedsQL ^TM^ Genetic Core Scales for Young Children (ages 5-7) | 0.9566 | 0.9233 | 0.9367 |
| PedsQL ^TM^ Genetic Core Scales for Children (ages 8-12) | 0.9407 | 0.9113 | 0.9297 |
| PedsQL ^TM^ Genetic Core Scales for Teens (ages 13-18) | 0.9469 | 0.9180 | 0.9376 |

**Supplementary Table 2.** Number of children and PedsQL score by disease (n=9,110)

| Disease | n (%) | Overall HRQoL | | |
| --- | --- | --- | --- | --- |
|  |  | Overall | Age 2-17 years | Age 1-23 months |
| *Endocrine, nutritional and metabolic diseases* |  | *Mean (SD)* | *Mean (SD)* | *Mean (SD)* |
| Phenylketonuria | 2435 (26.7%) | 70.24 (17.63) | 70.30 (18.11) | 70.05 (16.02) |
| Congenital adrenal hyperplasia (CAH) | 845 (9.3%) | 74.53 (16.08) | 77.65 (14.93) | 68.29 (16.51) |
| Albinism | 261 (2.9%) | 69.49 (17.48) | 69.83 (17.62) | 68.61 (17.22) |
| Mucopolysaccharidosis (MPS) | 218 (2.4%) | 32.39 (19.01) | 32.03 (18.85) | 57.78 (13.75) |
| Tetrahydrobiopterin deficiency | 207 (2.3%) | 61.49 (16.85) | 61.93 (17.70) | 60.44 (14.70) |
| Hepatolenticular degeneration (HLD); Wilson disease | 90 (1.0%) | 68.44 (21.88) | 68.44 (21.88) | NA |
| Gaucher disease (GD) | 81 (0.9%) | 50.04 (22.04) | 50.67 (22.47) | 43.29 (16.65) |
| Niemann-Pick disease (NPC) | 58 (0.6%) | 42.19 (24.08) | 41.38 (24.63) | 53.16 (11.53) |
| Glycogen storage disease type II (Pompe disease) | 38 (0.4%) | 45.83 (21.98) | 42.37 (20.47) | 64.28 (22.16) |
| Fabry disease | 19 (0.2%) | 59.00 (22.18) | 59.00 (22.18) | NA |
| Homozygous familial hypercholesterolemia (HoFH) | 8 (0.1%) | 61.68 (21.03) | 60.66 (22.50) | 68.89 (NA) |
| Kallmann syndrome (KS) | 2 (0.02%) | 64.05 (30.63) | 64.05 (30.63) | NA |
| *Diseases of the nervous system* |  |  |  |  |
| Duchenne Muscular Dystrophy (DMD) | 1216 (13.4%) | 44.58 (17.98) | 43.31 (17.29) | 63.70 (17.31) |
| Dravet syndrome (DS) | 328 (3.6%) | 38.36 (17.58) | 35.91 (17.08) | 52.34 (13.46) |
| Myasthenia gravis (MG) | 200 (2.2%) | 69.47 (17.95) | 69.34 (18.20) | 71.82 (13.23) |
| Spinal muscular atrophy (SMA) | 154 (1.7%) | 55.61 (15.34) | NA | 55.61 (15.34) |
| Multiple sclerosis (MS) | 12 (0.1%) | 64.01 (22.40) | 65.98 (24.22) | 54.17 (2.95) |
| Neuromyelitis optica spectrum disorder (NMOSD) | 12 (0.1%) | 62.43 (20.69) | 62.43 (20.69) | NA |
| Huntington's Disease (HD) | 3 (0.03%) | 19.44 (20.97) | 19.44 (20.97) | NA |
| *Congenital malformations, deformations and chromosomal abnormalities* | | | | |
| Tuberous sclerosis complex (TSC) | 856 (9.4%) | 49.14 (21.90) | 47.77 (22.63) | 56.12 (16.00) |
| Prader-Willi Syndrome (PWS) | 554 (6.1%) | 42.36 (16.33) | 39.20 (16.44) | 50.08 (13.21) |
| Inherited epidermolysis bullosa (EB) | 376 (4.1%) | 49.10 (22.35) | 47.00 (23.09) | 55.40 (18.73) |
| Marfan syndrome (MS) | 88 (1.0%) | 59.03 (17.80) | 58.84 (17.98) | 64.40 (12.58) |
| Osteogenesis imperfecta (OI) | 56 (0.6%) | 47.81 (17.59) | 46.81 (17.17) | 65.46 (18.91) |
| *Diseases of the blood and blood-forming organs and certain disorders involving the immune mechanism* | | | | |
| Hemophilia | 730 (8.0%) | 58.85 (18.99) | 57.75 (19.2) | 68.08 (14.11) |
| *Diseases of the musculoskeletal system and connective tissue* | | | | |
| Systemic sclerosis (SSc) | 149 (1.6%) | 68.78 (19.69) | 68.78 (19.69) | NA |
| *Neoplasms* |  |  |  |  |
| Langerhans cell histiocytosis (LCH) | 109 (1.2%) | 68.01 (20.23) | 68.39 (20.88) | 67.07 (18.78) |
| *Diseases of the circulatory system* |  |  |  |  |
| Idiopathic pulmonary arterial hypertension (IPAH) | 5 (0.1%) | 41.20 (19.66) | 41.20 (19.66) | NA |

NA: not available due to lack of case

**Supplementary Table 3.** Multivariable linear regression to identify factors associated with physical, psychosocial, and overall PedsQL scores (n=9,110)

| Variables | Physical HRQoL | |  | Psychosocial HRQoL | |  | Overall HRQoL | |
| --- | --- | --- | --- | --- | --- | --- | --- | --- |
| **Sex** | *Coeff (95% CI)* | *p-value* |  | *Coeff (95% CI)* | *p-value* |  | *Coeff (95% CI)* | *p-value* |
| Male | Ref. |  |  | Ref. |  |  | Ref. |  |
| Female | 3.25 (2.36, 4.13) | <0.001 |  | 1.55 (0.82, 2.28) | <0.001 |  | 2.21 (1.51, 2.91) | <0.001 |
| **Age of child**, years |  |  |  |  |  |  |  |  |
| <2 | Ref. |  |  | Ref. |  |  | Ref. |  |
| 2-4 | -2.07 (-3.27, -0.86) | <0.001 |  | -2.83 (-3.82, -1.83) | <0.001 |  | -2.53 (-3.49, -1.57) | <0.001 |
| 5-17 | -6.53 (-7.66, -5.41) | <0.001 |  | -7.34 (-8.26, -6.42) | <0.001 |  | -7.12 (-8.01, -6.22) | <0.001 |
| **Household registration** |  |  |  |  |  |  |  |  |
| Rural | —— |  |  | Ref. |  |  | Ref. |  |
| Urban | —— |  |  | 1.16 (0.48, 1.84) | <0.001 |  | 0.93 (0.18, 1.67) | 0.015 |
| **Parental education** |  |  |  |  |  |  |  |  |
| At least one parent below secondary | Ref. |  |  | —— |  |  | Ref. |  |
| Both secondary or above | 2.26 (1.34, 3.18) | <0.001 |  | —— |  |  | 1.28 (0.53, 2.04) | 0.001 |
| **Annual household income** |  |  |  |  |  |  |  |  |
| > CNY¥100,000 | Ref. |  |  | —— |  |  | —— |  |
| CNY¥50,000-100,000 | 1.31 (0.35, 2.26) | 0.007 |  | —— |  |  | —— |  |
| <CNY¥50,000 | 2.33 (1.03, 3.63) | <0.001 |  | —— |  |  |  |  |
| **Family Functioning** | 0.27 (0.25, 0.29) | <0.001 |  | 0.35 (0.34, 0.37) | <0.001 |  | 0.32 (0.31, 0.34) | <0.001 |
| **Dependence on assistive devices** |  |  |  |  |  |  |  |  |
| None | Ref. |  |  | Ref. |  |  | Ref. |  |
| Mild | -16.42 (-17.56, -15.29) | <0.001 |  | -10.65 (-11.58, -9.72) | <0.001 |  | -12.91 (-13.81, -12.01) | <0.001 |
| Substantial | -23.11 (-24.55, -21.68) | <0.001 |  | -12.06 (-13.24, -10.87) | <0.001 |  | -16.44 (-17.58, -15.30) | <0.001 |
| **Treatment** |  |  |  |  |  |  |  |  |
| Active | Ref. |  |  | Ref. |  |  | Ref. |  |
| Non-active | -3.44 (-4.42, -2.45) | <0.001 |  | -1.52 (-2.33, -0.71) | <0.001 |  | -2.34 (-3.12, -1.55) | <0.001 |
| **Diagnostic delay** |  |  |  |  |  |  |  |  |
| None | Ref. |  |  | Ref. |  |  | Ref. |  |
| 1-2 years | -5.24 (-6.69, -3.80) | <0.001 |  | -4.46 (-5.65, -3.26) | <0.001 |  | -4.89 (-6.04, -3.75) | <0.001 |
| ≥3 years | -5.49 (-8.10, -2.88) | <0.001 |  | -6.71 (-8.86, -4.56) | <0.001 |  | -6.13 (-8.20, -4.05) | <0.001 |
| **Comorbidity** |  |  |  |  |  |  |  |  |
| None | Ref. |  |  | Ref. |  |  | Ref. |  |
| 1 | -4.26 (-5.32, -3.19) | <0.001 |  | -6.38 (-7.26, -5.51) | <0.001 |  | -5.60 (-6.44, -4.76) | <0.001 |
| ≥2 | -10.18 (-11.45, -8.91) | <0.001 |  | -14 (-15.05, -12.96) | <0.001 |  | -12.6 (-13.6, -11.59) | <0.001 |
| **Disease category** |  |  |  |  |  |  |  |  |
| Endocrine, nutritional and metabolic diseases | Ref. |  |  | Ref. |  |  | Ref. |  |
| Diseases of  musculoskeletal system | -16.17 (-17.37, -14.97) | <0.001 |  | -6.17 (-7.15, -5.20) | <0.001 |  | -9.88 (-10.82, -8.94) | <0.001 |
| Congenital malformations, deformations and chromosomal abnormalities | -11.49 (-12.64, -10.34) | <0.001 |  | -8.86 (-9.80, -7.91) | <0.001 |  | -9.9 (-10.81, -8.98) | <0.001 |
| Others | -5.79 (-7.21, -4.36) | <0.001 |  | -0.31 (-1.48, 0.86) | 0.605 |  | -2.27 (-3.40, -1.15) | <0.001 |

CNY¥1=USD$0.14. —— Non-significant association

**Supplementary Table 4.** Multivariable linear regression to identify factors associated with physical, psychosocial, and overall PedsQL scores (n=9,110)

| Variables | Physical HRQoL | |  | Psychosocial HRQoL | |  | Overall HRQoL | |
| --- | --- | --- | --- | --- | --- | --- | --- | --- |
| **Sex** | *Coeff (95% CI)* | *p-value* |  | *Coeff (95% CI)* | *p-value* |  | *Coeff (95% CI)* | *p-value* |
| Male | Ref. |  |  | Ref. |  |  | Ref. |  |
| Female | 3.25 (2.38, 4.13) | <0.001 |  | 1.56 (0.85, 2.28) | <0.001 |  | 2.22 (1.53, 2.91) | <0.001 |
| **Age of child**, years |  |  |  |  |  |  |  |  |
| <2 | Ref. |  |  | Ref. |  |  | Ref. |  |
| 2-4 | -2.47 (-3.67, -1.28) | <0.001 |  | -3.32 (-4.29, -2.34) | <0.001 |  | -2.98 (-3.92, -2.04) | <0.001 |
| 5-17 | -6.67 (-7.78, -5.56) | <0.001 |  | -7.42 (-8.32, -6.52) | <0.001 |  | -7.22 (-8.1, -6.35) | <0.001 |
| **Household registration** |  |  |  |  |  |  |  |  |
| Rural | —— |  |  | Ref. |  |  | Ref. |  |
| Urban | —— |  |  | 0.93 (0.26, 1.59) | <0.001 |  | 0.81 (0.08, 1.54) | 0.029 |
| **Parental education** |  |  |  |  |  |  |  |  |
| At least one parent below secondary | Ref. |  |  | —— |  |  | Ref. |  |
| Both secondary or above | 2.11 (1.21, 3.02) | <0.001 |  | —— |  |  | 1.08 (0.34, 1.82) | 0.004 |
| **Annual household income** |  |  |  |  |  |  |  |  |
| > CNY¥100,000 | Ref. |  |  | —— |  |  | —— |  |
| CNY¥50,000-100,000 | 1.21 (0.26, 2.15) | 0.026 |  | —— |  |  | —— |  |
| <CNY¥50,000 | 1.99 (0.71, 3.28) | 0.009 |  | —— |  |  |  |  |
| **Parental HRQoL** | 0.32 (0.3, 0.34) | <0.001 |  | 0.41 (0.39, 0.43) | <0.001 |  | 0.38 (0.36, 0.39) | <0.001 |
| **Dependence on assistive devices** |  |  |  |  |  |  |  |  |
| None | Ref. |  |  | Ref. |  |  | Ref. |  |
| Mild | -16.18 (-17.31, -15.06) | <0.001 |  | -10.4 (-11.31, -9.48) | <0.001 |  | -12.66 (-13.54, -11.78) | <0.001 |
| Substantial | -22.73 (-24.15, -21.31) | <0.001 |  | -11.62 (-12.78, -10.46) | <0.001 |  | -16.03 (-17.15, -14.91) | <0.001 |
| **Treatment** |  |  |  |  |  |  |  |  |
| Active | Ref. |  |  | Ref. |  |  | Ref. |  |
| Non-active | -3.64 (-4.61, -2.66) | <0.001 |  | -1.76 (-2.56, -0.97) | <0.001 |  | -2.55 (-3.32, -1.79) | <0.001 |
| **Diagnostic delay** |  |  |  |  |  |  |  |  |
| None | Ref. |  |  | Ref. |  |  | Ref. |  |
| 1-2 years | -5.2 (-6.63, -3.77) | <0.001 |  | -4.45 (-5.61, -3.28) | <0.001 |  | -4.86 (-5.99, -3.74) | <0.001 |
| ≥3 years | -5.06 (-7.65, -2.48) | <0.001 |  | -6.14 (-8.25, -4.04) | <0.001 |  | -5.64 (-7.68, -3.61) | <0.001 |
| **Comorbidity** |  |  |  |  |  |  |  |  |
| None | Ref. |  |  | Ref. |  |  | Ref. |  |
| 1 | -4 (-5.05, -2.95) | <0.001 |  | -6.08 (-6.94, -5.22) | <0.001 |  | -5.33 (-6.15, -4.5) | <0.001 |
| ≥2 | -9.87 (-11.12, -8.62) | <0.001 |  | -13.69 (-14.71, -12.67) | <0.001 |  | -12.29 (-13.27, -11.3) | <0.001 |
| **Disease category** |  |  |  |  |  |  |  |  |
| Endocrine, nutritional and metabolic diseases | Ref. |  |  | Ref. |  |  | Ref. |  |
| Diseases of  musculoskeletal system | -15.31 (-16.5, -14.12) | <0.001 |  | -5.16 (-6.12, -4.2) | <0.001 |  | -8.94 (-9.86, -8.01) | <0.001 |
| Congenital malformations, deformations and chromosomal abnormalities | -11.36 (-12.5, -10.22) | <0.001 |  | -8.74 (-9.67, -7.82) | <0.001 |  | -9.78 (-10.68, -8.89) | <0.001 |
| Others | -5.72 (-7.13, -4.31) | <0.001 |  | -0.22 (-1.36, 0.93) | 0.709 |  | -2.21 (-3.31, -1.1) | <0.001 |

CNY¥1=USD$0.14. —— Non-significant association

**Supplementary Table 5.** Subgroup analysis of factors associated with physical, psychosocial, and overall PedsQL scores by children/adolescents aged 2-17 years and infants aged 1-23 months

(A) Children/adolescents aged 2-17 years (n=7,293)

| Variables | Physical HRQoL | |  | Psychosocial HRQoL | |  | Overall HRQoL | |
| --- | --- | --- | --- | --- | --- | --- | --- | --- |
|  | *Coeff (95% CI)* | *p-value* |  | *Coeff (95% CI)* | *p-value* |  | *Coeff (95% CI)* | *p-value* |
| **Sex** |  |  |  |  |  |  |  |  |
| Male | Ref. |  |  | Ref. |  |  | Ref. |  |
| Female | 3.29 (2.26, 4.31) | <0.001 |  | 1.57 (0.77, 2.38) | <0.001 |  | 2.23 (1.45, 3.02) | <0.001 |
| **Age of child**, years |  |  |  |  |  |  |  |  |
| 2-4 | Ref. |  |  | Ref. |  |  | Ref. |  |
| 5-7 | -0.89 (-2.07, 0.30) | 0.14 |  | -3.53 (-4.46, -2.59) | <0.001 |  | -2.76 (-3.66, -1.85) | <0.001 |
| 8-12 | -6.66 (-7.90, -5.43) | <0.001 |  | -4.08 (-5.06, -3.10) | <0.001 |  | -5.15 (-6.09, -4.20) | <0.001 |
| 13-17 | -6.99 (-8.79, -5.19) | <0.001 |  | -4.66 (-6.08, -3.24) | <0.001 |  | -5.68 (-7.05, -4.31) | <0.001 |
| **Household registration** |  |  |  |  |  |  |  |  |
| Rural | Ref. | 0.02 |  | —— |  |  | —— |  |
| Urban | 1.25 (0.17, 2.33) |  |  | —— |  |  | —— |  |
| **Parental education** |  |  |  |  |  |  |  |  |
| At least one parent below secondary | Ref. |  |  | Ref. |  |  | Ref. |  |
| Both secondary or above | 1.99 (0.90, 3.09) | <0.001 |  | 0.75 (0.01, 1.50) | 0.04 |  | 1.43 (0.70, 2.16) | <0.001 |
| **Parental HRQoL** | 0.25 (0.21, 0.28) | <0.001 |  | 0.31 (0.28, 0.34) | <0.001 |  | 0.28 (0.25, 0.31) | <0.001 |
| **Family Functioning** | 0.11 (0.08, 0.14) | <0.001 |  | 0.16 (0.13, 0.18) | <0.001 |  | 0.14 (0.11, 0.17) | <0.001 |
| **Dependence on assistive devices** |  |  |  |  |  |  |  |  |
| None | Ref. |  |  | Ref. |  |  | Ref. |  |
| Mild | -17.43 (-18.7, -16.16) | <0.001 |  | -10.99 (-11.99, -10.00) | <0.001 |  | -13.50 (-14.47, -12.53) | <0.001 |
| Substantial | -25.84 (-27.51, -24.16) | <0.001 |  | -12.84 (-14.16, -11.53) | <0.001 |  | -18.04 (-19.32, -16.76) | <0.001 |
| **Treatment** |  |  |  |  |  |  |  |  |
| Active | Ref. |  |  | Ref. |  |  | Ref. |  |
| Non-active | -4.58 (-5.68, -3.47) | <0.001 |  | -2.32 (-3.19, -1.45) | 0.06 |  | -3.23 (-4.08, -2.39) | <0.001 |
| **Diagnostic delay** |  |  |  |  |  |  |  |  |
| None | Ref. |  |  | Ref. |  |  | Ref. |  |
| 1-2 years | -5.36 (-6.93, -3.80) | <0.001 |  | -4.99 (-6.22, -3.76) | <0.001 |  | -5.14 (-6.34, -3.95) | <0.001 |
| ≥3 years | -3.22 (-5.91, -0.53) | 0.03 |  | -5.56 (-7.68, -3.43) | <0.001 |  | -4.50 (-6.56, -2.45) | <0.001 |
| **Comorbidity** |  |  |  |  |  |  |  |  |
| None | Ref. |  |  | Ref. |  |  | Ref. |  |
| 1 | -4.19 (-5.40, -2.98) | <0.001 |  | -6.08 (-7.03, -5.13) | <0.001 |  | -5.43 (-6.36, -4.50) | <0.001 |
| ≥2 | -9.49 (-10.89, -8.09) | <0.001 |  | -13.21 (-14.31, -12.11) | <0.001 |  | -11.86 (-12.93, -10.78) | <0.001 |
| **Diagnosis** |  |  |  |  |  |  |  |  |
| Recent | —— |  |  | Ref. |  |  | —— |  |
| Non-recent | —— |  |  | -1.13 (-2.2, -0.05) | 0.04 |  | —— |  |
| **Disease category** |  |  |  |  |  |  |  |  |
| Endocrine, nutritional and metabolic diseases | Ref. |  |  | Ref. |  |  | Ref. |  |
| Diseases of musculoskeletal system | -17.54 (-18.87, -16.21) | <0.001 |  | -6.84 (-7.92, -5.76) | <0.001 |  | -10.71 (-11.72, -9.69) | <0.001 |
| Congenital malformations, deformations and chromosomal abnormalities | -12.53 (-13.85, -11.21) | <0.001 |  | -9.80 (-10.84, -8.76) | <0.001 |  | -10.89 (-11.90, -9.88) | <0.001 |
| Others | -6.62 (-8.19, -5.06) | <0.001 |  | -1.14 (-2.37, 0.10) | 0.07 |  | -3.02 (-4.21, -1.82) | <0.001 |

—— Non-significant association

(B) infants aged 1-23 months (n=1,817)

| Variables | Physical HRQoL | |  | Psychosocial HRQoL | |  | Overall HRQoL | |
| --- | --- | --- | --- | --- | --- | --- | --- | --- |
| **Age of child**, months | *Coeff (95% CI)* | *p-value* |  | *Coeff (95% CI)* | *p-value* |  | *Coeff (95% CI)* | *p-value* |
| 1-12 | —— |  |  | Ref. |  |  | —— |  |
| 13-23 | —— |  |  | 2.78 (1.45, 4.12) | <0.001 |  | —— |  |
| **Household registration** |  |  |  |  |  |  |  |  |
| Rural | —— |  |  | -Ref. |  |  | Ref. |  |
| Urban | —— |  |  | 1.81 (0.50, 3.13) | 0.007 |  | 1.53 (0.31, 2.75) | 0.01 |
| **Parental marital status** |  |  |  |  |  |  |  |  |
| Married | —— |  |  | Ref. |  |  | —— |  |
| Separated | —— |  |  | -3.70 (-6.10, -1.30) | 0.003 |  | —— |  |
| **Parental HRQoL** | 0.26 (0.20, 0.31) | <0.001 |  | 0.24 (0.20, 0.29) | <0.001 |  | 0.27 (0.22, 0.31) | <0.001 |
| **Family functioning** | 0.11 (0.05, 0.16) | <0.001 |  | 0.09 (0.04, 0.13) | <0.001 |  | 0.10 (0.06, 0.14) | <0.001 |
| **Dependence on assistive devices** |  |  |  |  |  |  |  |  |
| None | Ref. |  |  | Ref. |  |  | Ref. |  |
| Mild | -9.54 (-11.99, -7.08) | <0.001 |  | -5.70 (-7.70, -3.71) | <0.001 |  | -6.29 (-8.15, -4.44) | <0.001 |
| Substantial | -15.23 (-17.92, -12.53) | <0.001 |  | -8.50 (-10.69, -6.31) | <0.001 |  | -9.29 (-11.32, -7.26) | <0.001 |
| **Comorbidity** |  |  |  |  |  |  |  |  |
| None | Ref. |  |  | Ref. |  |  | Ref. |  |
| 1 | -4.16 (-6.26, -2.05) | <0.001 |  | -4.28 (-5.99, -2.56) | <0.001 |  | -4.85 (-6.44, -3.26) | <0.001 |
| ≥2 | -6.24 (-9.20, -3.29) | <0.001 |  | -10.43 (-12.83, -8.03) | <0.001 |  | -10.33 (-12.56, -8.10) | <0.001 |
| **Disease category** |  |  |  |  |  |  |  |  |
| Endocrine, nutritional and metabolic diseases | Ref. |  |  | Ref. |  |  | Ref. |  |
| Diseases of musculoskeletal system | -12.07 (-14.57, -9.57) | <0.001 |  | 0.02 (-2.04, 2.09) | 0.06 |  | -2.19 (-4.08, -0.30) | <0.001 |
| Congenital malformations, deformations and chromosomal abnormalities | -17.08 (-19.30, -14.87) | <0.001 |  | -2.15 (-3.97, -0.34) | <0.001 |  | -6.86 (-8.54, -5.19) | <0.001 |
| Others | -5.77 (-9.25, -2.30) | 0.001 |  | 1.46 (-1.37, 4.30) | 0.58 |  | 0.81 (-1.81, 3.44) | <0.001 |

—— Non-significant association
